# Donor plasma mitochondrial DNA is associated with antibody-mediated rejection in renal allograft recipients

**DOI:** 10.18632/aging.202654

**Published:** 2021-03-10

**Authors:** Fei Han, Qipeng Sun, Zhengyu Huang, Heng Li, Maolin Ma, Tao Liao, Zihuang Luo, Lingling Zheng, Nana Zhang, Nan Chen, Liangqing Hong, Ning Na, Qiquan Sun

**Affiliations:** 1 Organ Transplantation Research Institution, Division of Kidney Transplantation, The Third Affiliated Hospital, Sun Yat-Sen University, Guangzhou, China; 2 Department of Medical Statistics and Epidemiology, School of Public Health, Sun Yat-Sen University, Guangzhou, China; 3 Department of Pathology, The Third Affiliated Hospital, Sun Yat-Sen University, Guangzhou, China; 4 Laboratory of Cancer Biomarkers and Liquid Biopsy, Henan University, Kaifeng, China

**Keywords:** kidney transplantation, mitochondrial DNA, antibody-mediated rejection

## Abstract

We previously showed that donor plasma mitochondrial DNA (dmtDNA) levels were correlated with renal allograft function. The aim of the current study was to determine whether dmtDNA levels are associated with the occurrence of antibody-mediated rejection (ABMR). This is a retrospective open cohort study comprised of 167 donors and 323 recipients enrolled from January 2015 to December 2017. We quantified the mtDNA level present in donor plasma using quantitative real-time polymerase chain reaction. The average plasma dmtDNA level in the acute rejection (AR) group was higher than that of the control group (0.156 versus 0.075, p<0.001). Multivariate logistic regression analysis showed that dmtDNA levels were also significantly associated with AR (OR=1.588, 95% CI 1.337-4.561, p<0.001). When the dmtDNA level was >0.156, the probability of AR was 62.9%. The plasma dmtDNA level in the ABMR group was significantly higher than that of the T cell-mediated rejection group (0.185 versus 0.099, p=0.032). The area under the receiver operating characteristic curve of dmtDNA for prediction of ABMR was as high as 0.910 (95% CI 0.843-0.977). We demonstrated that plasma dmtDNA was an independent risk factor for ABMR, which is valuable in organ evaluation. dmtDNA level is a possible first predictive marker for ABMR.

## INTRODUCTION

Kidney transplantation is the optimal therapy for end-stage renal disease (ESRD) [1]. Immunosuppressive therapy after kidney transplantation has significantly improved allograft survival time [2]. However, the mean time of deceased-donor transplants was only 8.8 years [3]. Antibody-mediated rejection (ABMR) considerably increases allograft mortality, and remains the main reason for the failure of kidney transplants, and the return of kidney transplant recipients to dialysis [4, 5]. However, no reliable metrics have been identified that can predict incidence of ABMR in kidney allograft recipients prior to transplantation.

Mitochondrial DNA (mtDNA) is double-stranded DNA located in the mitochondrial matrix that encodes several proteins [6, 7]. When mitochondria are damaged, mtDNA will fragment, and then go into the cytosol and subsequently enter the systemic circulation [8]. It was reported that mtDNA a predictive biomarker of acute kidney injury (AKI) [9, 10]. Ischemic-reperfusion injury causes the release of mtDNA, which can elicit an immune response [11]. Studies have shown that mtDNA can induce cell-mediated immune rejection after kidney transplantation, mtDNA-derived mHAg plays a role in rejection [12]. In our previous study, donor plasma mitochondrial DNA (dmtDNA) level was associated with posttransplant renal allograft function [13]. However, no results were found referring to the relationship between dmtDNA levels and acute rejection (AR) in renal allograft recipients.

In this study, we assessed the association between dmtDNA levels and AR based on the findings of our previous studies. We hypothesized that higher dmtDNA levels are associated with greater risk of AR post-transplantation.

## RESULTS

### Study cohort

**Figure 1:**
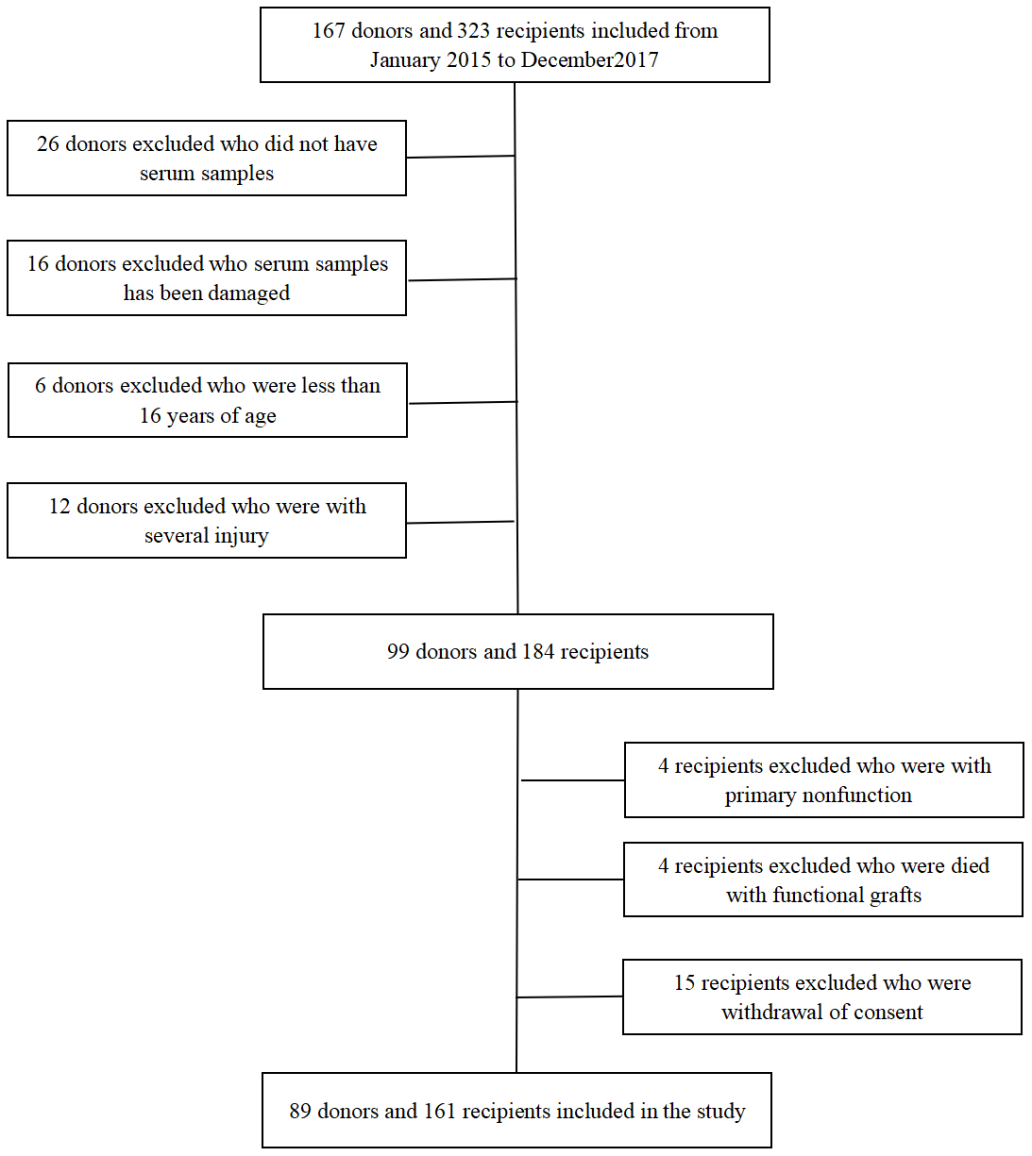
**Flow chart for kidney donors and recipients enrolled in the study.**

The study flowchart was showed in Figure 1. One hundred and sixty-seven donors and 323 recipients were included. According to the exclusion criteria, 89 donors and 161 recipients remained. The basic characteristics of donors and recipients are shown in Tables 1, 2.

**Table 1:** Deceased-donor characteristics, stratified by AR.

**Characteristic**	**All(n=161)**	**Normal recipient(n=143)**	**AR(n=18)**	***P* value**
**Age (years)**	43.1±12.6	43.1±13.1	42.1±9.7	0.75
**Male sex**	109(67.7)	98(68.5)	11(61.1)	0.53
**BMI (kg/m**^2^**)**	23[21.25,25.0]	23.0[21.2,25.0]	23.2[21.8,23.9]	0.934
**Hypertension**	67(41.6)	56(39.2)	11(61.1)	0.075
**Cause of death**				0.313
Head trauma	52(32.3)	47(32.9)	5(27.8)	
Stroke	64(39.8)	54(37.8)	10(55.6)	
Other	45(27.9)	42(29.3)	3(16.6)	
**Admission SCr, mg/dl**	0.77[0.32,1.40]	0.68[0.32,1.39]	1.27[1.03,1.91]	0.007
**Terminal SCr, mg/dl**	1.69[1.06,3.04]	1.64[1.04,2.98]	2.48[2.25,3.99]	0.003
**Extended criteria donor**	53(32.9)	49(34.2)	4(22.2)	0.305
**Use of any vasoactive drugs**	142(88.2)	127(88.8)	15(83.3)	0.497
**No. of vasoactive agents used**	2[1,2]	2[1,2]	2[1,2.25]	0.302
**Plasma dmtDNA**	0.062[0.028,0.127]	0.055[0.027,0.109]	0.164[0.097,0.195]	0.001

Continuous variables according to Shapiro test, if P>0.05 the data are expressed as mean±SD, otherwise, the data are expressed as median[P25,P75]; Categorical variables are described by total numbers and percentages. Continuous variables were compared using ANOVA or Kruskal-Wallis tests and categorical variables were compared using the c2 test and Fisher’s exact test.

Abbreviations: BMI: body mass index; SCr: serum creatinine; dmtDNA: donor mitochondrial DNA; ANOVA: analysis of variance; AR: acute rejection.

**Table 2:** Recipient characteristics, stratified by AR.

**Characteristic**	**All(n=161)**	**Normal recipient(n=143)**	**AR(n=18)**	***P* value**
**Age, years**	43.9±11.29	44.08±11.49	42.5±9.69	0.577
**Male sex**	106(65.8)	94(65.7)	12(66.7)	0.937
**Cause of ESRD**				0.001
Hypertension	21(13.1)	12(8.4)	9(50)	
Diabetes	15(9.3)	13(9.1)	2(11.1)	
GN	30(18.6)	27(18.9)	3(16.7)	
PKD	5(3.1)	5(3.5)	0(0)	
Others	90(55.9)	86(60.1)	4(22.2)	
**Mode of dialysis**				0.115
HD	112(69.6)	98(68.5)	14(77.8)	
PD	47(29.2)	44(30.8)	3(16.7)	
HD+PD	2(1.2)	1(0.7)	1(5.5)	
**Dialysis duration, mo**	14[5,26]	14[5,26]	13.5[6.5,29.25]	0.569
**Warm ischemia time, min**	10[5,12]	13[10,16]	12[10,14]	0.931
**Cold ischemia time, h**	4[2,6]	6[2,6]	5[3,7.25]	0.117
**Number of HLA mismatches**	3[2,4]	3[2,4]	2[2,3]	0.057
**Panel reactive antibody**				0.884
0%	123(76.4)	109(76.2)	14(77.8)	
1%-10%	38(23.6)	34(23.8)	4(22.2)	
**Induction regimen**				0.820
ATG	131(81.4)	116(81.1)	15(83.3)	
Basiliximab	30(18.6)	27(18.9)	3(16.7)	
**CNI**				0.601
Cyclosporin	30(18.6)	26(18.1)	4(22.2)	
Tacrolimus	110(68.3)	99(69.3)	11(61.1)	
**mTOR inhibitor**	21(13.1)	18(12.6)	3(16.7)	0.628
**Steroid**	161	143	18	
**Number of graft loss**	20(12.4)	10(6.9)	10(55.6)	0.001

Continuous variables according to the Shapiro test, if *P* > 0.05, the data are expressed as mean ± SD; otherwise data are expressed as median[P25,P75]; categorical variables are described by numbers and percentages (%). Continuous variables were compared using ANOVA or Kruskal–Wallis tests, categorical variables were compared using the χ2 test and Fisher’s exact test.

ESRD, end-stage renal disease; GN, glomerulonephritis; PKD, polycystic kidney disease; HD, hemodialysis; PD, peritoneal dialysis; HLA, human leukocyte antigen; ANOVA, analysis of variance; CNI, calcineurin inhibitor. AR: acute rejection.

### Plasma dmtDNA level is correlated with post-transplant DGF

**Figure 2:**
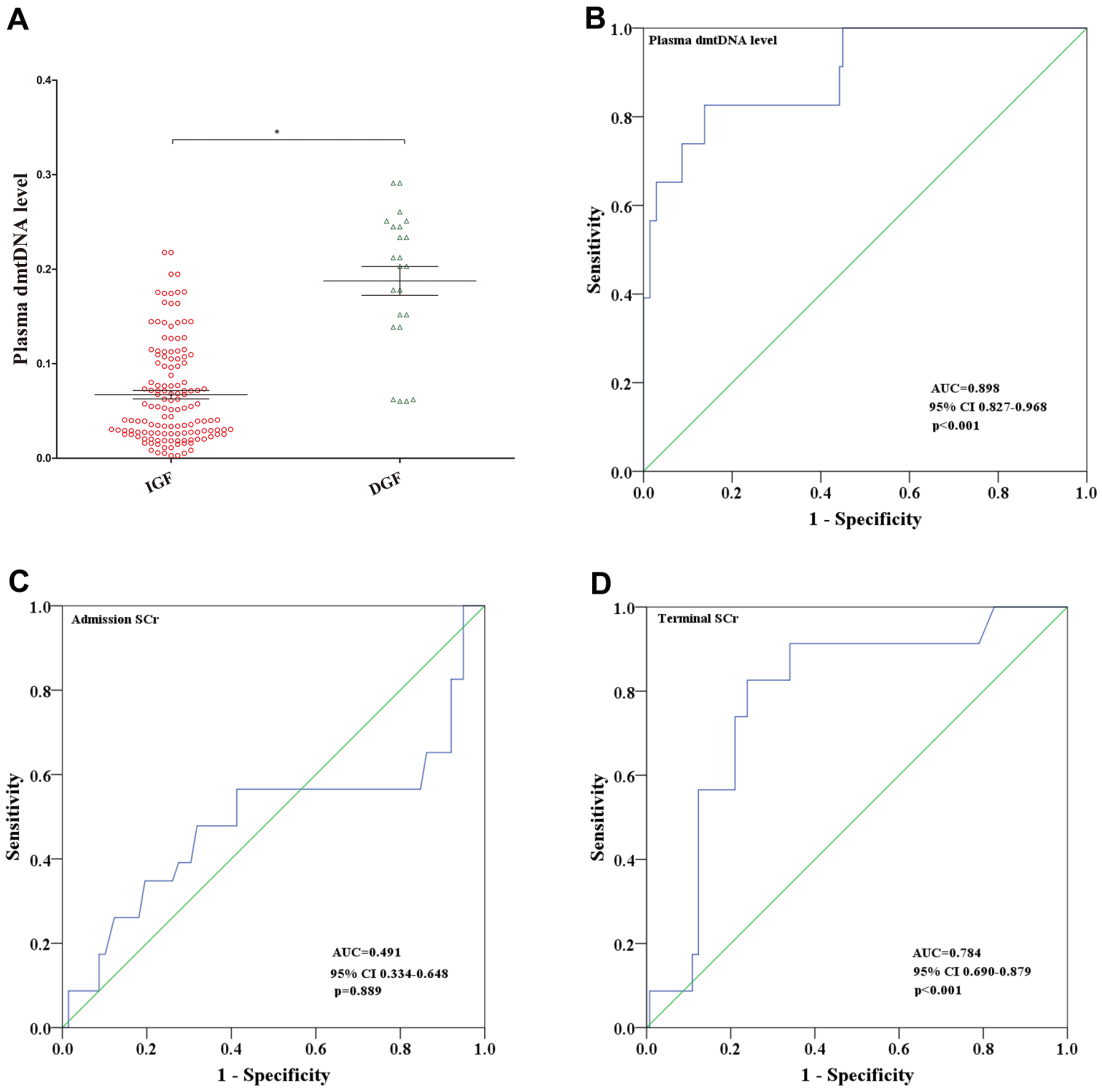
(**A**) Distribution of plasma dmtDNA in the delayed graft function (DGF) (n=23) and immediate graft function (IGF) (n=138) groups. (**B**–**D**) The receiver operating characteristic (ROC) curves of plasma dmtDNA (**B**), donor admission serum creatinine levels (**C**), and donor terminal serum creatinine levels (**D**).

Of the 161 recipients, 23 (14.3%) recipients developed DGF. The average dmtDNA level in the DGF group was significantly higher than that in the IGF group (0.187 versus 0.067, p<0.001) (Figure 2A). The area under the receiver operating characteristic (ROC) curve of dmtDNA for the prediction score was 0.898 (95% confidence interval [CI] 0.827-0.968, p<0.001). The sensitivity and specificity of the prediction scores value were 82.6% and 86.2%, respectively based on the optimal prediction score cutoff value ≥0.133 (Figure 2B). When the dmtDNA level was >0.133, the probability of DGF in the recipient was 50%. The diagnostic performance of the dmtDNA level was higher than that of the donor admission SCr level (AUC=0.491, 95% CI 0.334-0.648, p=0.899) and the terminal SCr level (AUC=0.784, 95% CI 0.690-0.879, p<0.001) (Figure 2C, 2D).

### Plasma dmtDNA level is correlated with acute rejection

**Figure 3:**
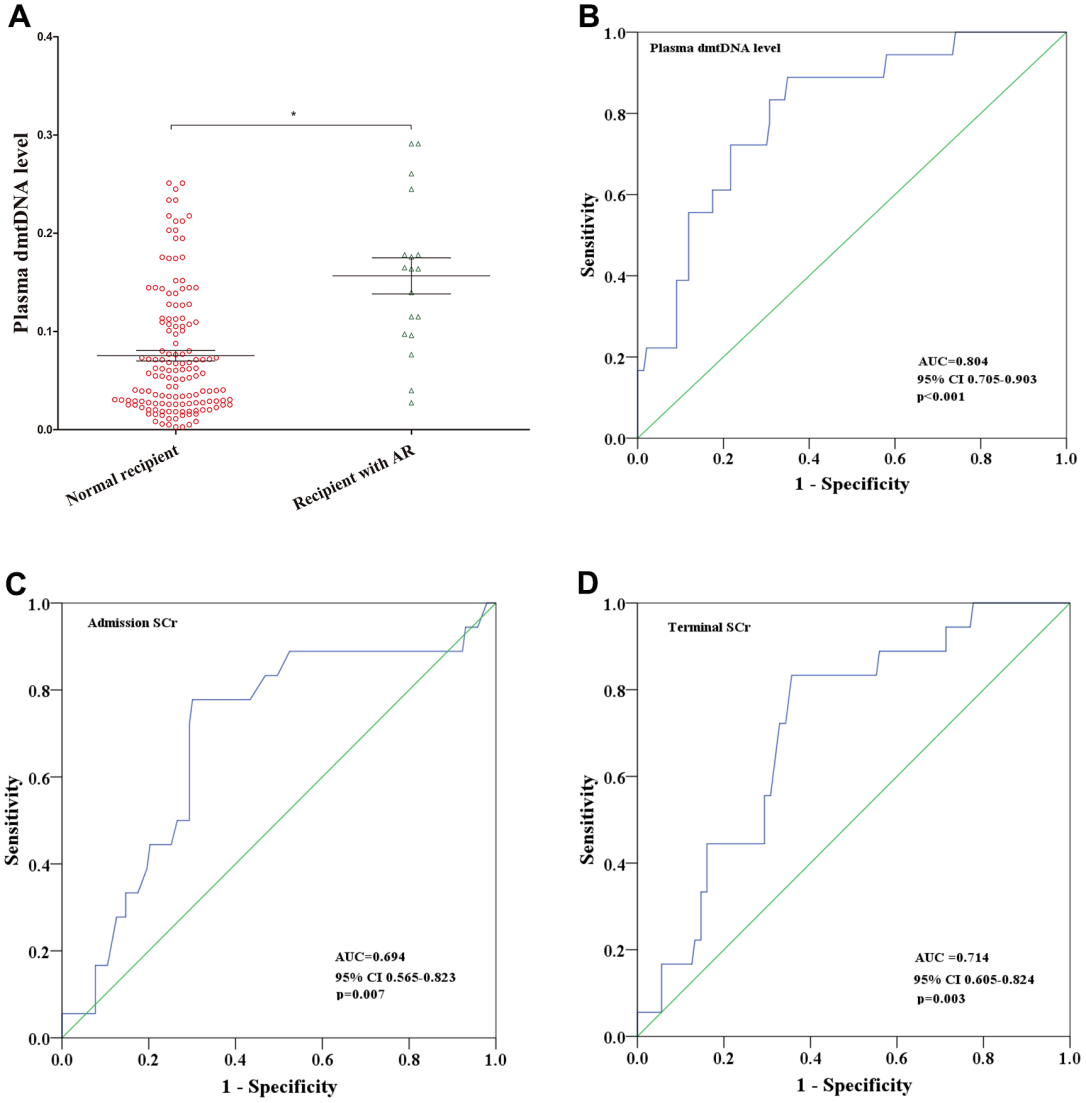
(**A**) Distribution of plasma dmtDNA in the normal recipient (n=143) and acute rejection (AR) (n=18) groups. (**B**–**D**) The receiver operating characteristic (ROC) curves of plasma dmtDNA (**B**), donor admission serum creatinine levels (**C**), and donor terminal serum creatinine levels (**D**).

A total of 18 (11.2%) recipients had AR. The level of dmtDNA in AR group was higher than normal group (0.156 versus 0.075, p<0.001) (Figure 3A). When the dmtDNA was > 0.0748, the probability of AR was 23.5%. However, if the dmtDNA level was >0.156, the probability of AR was 62.9%. Logistic regression analysis to evaluate the relationship between plasma dmtDNA and acute rejection (AR) in Table 3. dmtDNA level and induction therapy with anti-thymocyte globulin were correlated with AR (odds ratio (OR)=4.522, 95% CI3.041-6.934, p<0.001; OR=0.041, 95% CI 0.002-1.072, p=0.057, respectively). A multivariate logistic regression analysis showed that the plasma dmtDNA level was also correlated with AR (OR=1.588, 95% CI 1.337-4.561, p<0.001).

**Table 3:** Univariate and multivariate logistic regression analyses for the predictors of AR.

	**Univariate**				**Multivariate**		
	**OR**	**95%CI**	**P value**		**OR**	**95%CI**	**P value**
**Donor age (years)**	0.980	0.926-1.037	0.488				
**Donor sex (women)**	0.304	0.058-1.591	0.158				
**Donor BMI (kg/m**^2^**)**	0.866	0.663-1.132	0.293				
**Donor cause of death**	0.755	0.367-1.556	0.447				
**Donor terminal SCr (mg/ml)**	1.448	0.878-2.388	0.147				
**Warm ischemia time (min)**	0.943	0.830-1.072	0.372				
**Cold ischemia time (h)**	1.019	0.854-1.216	0.832				
**Induction therapy (ATG)**	0.041	0.002-1.072	0.057				
**Recipient age (years)**	0.987	0.930-1.048	0.675				
**Recipient sex (women)**	0.656	0.167-2.585	0.656				
**Mode of dialysis**	1.072	0.256-4.487	0.924				
**Duration of dialysis before transplantation (mo)**	1.014	0.991-1.039	0.240				
**HLA mismatch**	0.614	0.337-1.119	0.111				
**PRA**	5.599	0.312-10.612	0.243				
**Plasma dmtDNA**	4.522	3.041-6.934	0.001		1.588	1.337-4.561	0.001

Multivariate logistic regression analysis was performed with a backward selection procedure.

Abbreviation: BMI: body mass index; SCr: serum creatinine; CI: confidence interval; OR: odds ratio. HLA, human leukocyte antigen; PRA: panel reactive antibody; ATG: Anti-thymocyte globulin; SD: Standard deviation.

The diagnostic value of dmtDNA levels, admission SCr levels, and terminal SCr levels for prediction of AR were assessed using ROC curve analysis. The ROC curve analysis indicated that plasma dmtDNA levels could diagnose AR (AUC=0.804, 95% CI 0.705-0.903, p<0.001) when compared with admission SCr and terminal SCr levels (AUC=0.694, 95% CI 0.565-0.823, p=0.007; AUC=0.714, 95% CI 0.605-0.824, p=0.003) (Figure 3C, 3D). The maximum Youden-J indices showed that the sensitivity was 88.9%, and specificity was 69%, with the cutoff value for the dmtDNA level ≥0.0748.

### Plasma dmtDNA levels show a good diagnostic value for ABMR

**Figure 4:**
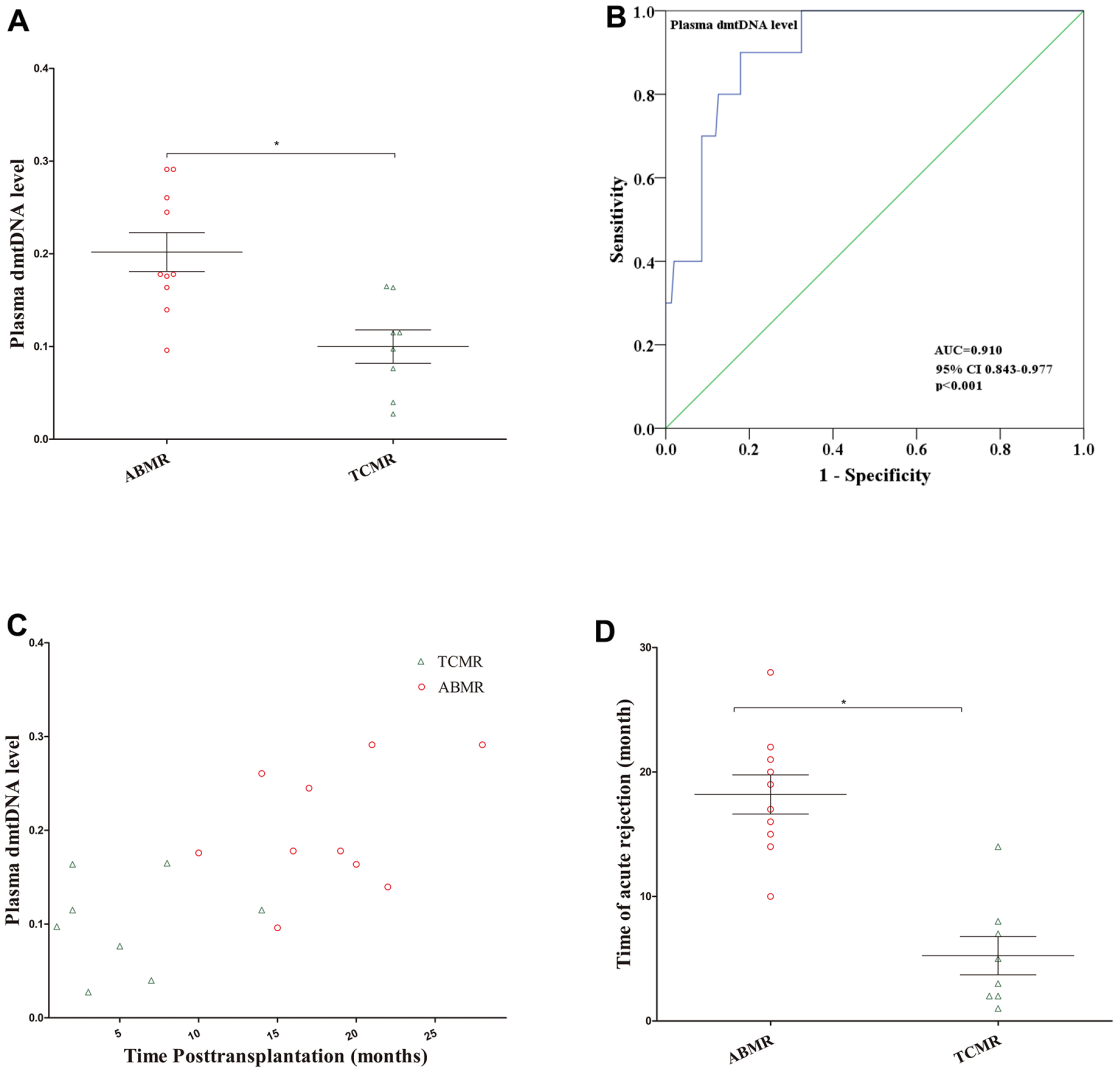
(**A**) Distribution of plasma dmtDNA in the antibody-mediated rejection (ABMR) (n=10) and T cell-mediated rejection (TCMR) (n=8) groups. (**B**) The receiver operating characteristic curve indicating the different donor performance characteristics between patients with ABMR and non-ABMR. (**C**) The time point of diagnosis of ABMR and TCMR post-transplantation (months). (**D**) The time point of patients with ABMR was significantly higher than that in TCMR group.

Of the 18 recipients diagnosed with AR, 10 (55.6%) recipients were diagnosed with ABMR, 8 of 10 recipients were DSA positive, and 8 (44.4%) recipients were diagnosed with TCMR. dmtDNA level in the ABMR group was significantly higher than TCMR group (0.185 versus 0.099, p=0.032) (Figure 4A). We then conducted ROC curve analysis to obtain the prediction score for dmtDNA levels with regard to ABMR, AUC=0.910 (95% CI 0.843-0.977, p<0.001) (Figure 4B). According to the optimal prediction score cutoff value for the dmtDNA level ≥0.139, the sensitivity and specificity were 92% and 82.1%, respectively.

Additionally, we found that the time to diagnose ABMR post-transplantation is later than that of TCMR. Most ABMR occurs one year after transplantation, but most TCMR occurs within one year (Figure 4C). The average time for 10 recipients to be diagnosed with ABMR was 18.2 months, while the average time for 8 patients to be diagnosed TCMR was 5.25 months (p<0.001) (Figure 4D).

### Plasma dmtDNA level is correlated with allograft survival

**Figure 5:**
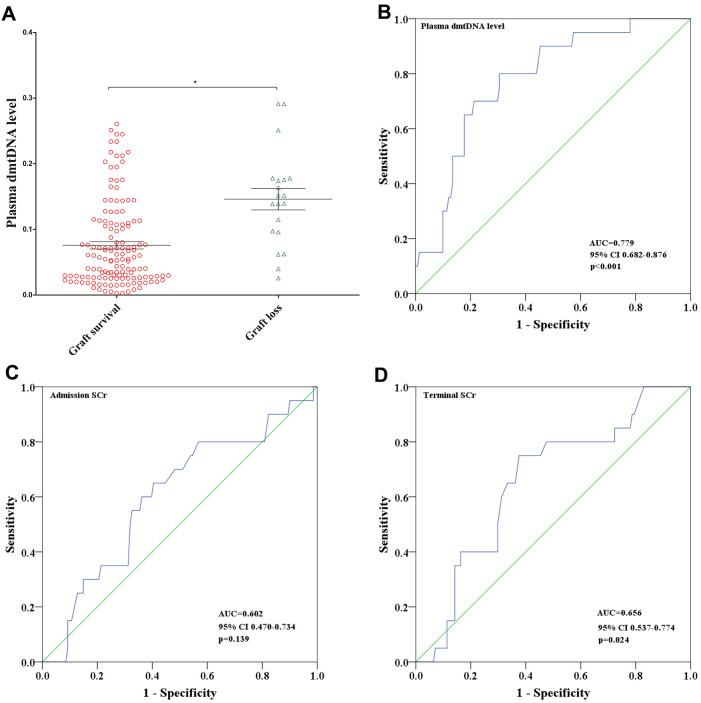
(**A**) Distribution of plasma dmtDNA in graft survival (n=141) and graft loss (n=20) groups. (**B**–**D**) The receiver operating characteristic (ROC) curves of plasma dmtDNA (**B**), donor admission serum creatinine levels (**C**), and donor terminal serum creatinine levels (**D**).

The average follow-up time for the 161 recipients was 44 [38,49] months. Of these 161 recipients, no one lost follow-up, and 20 of them experienced graft loss. dmtDNA level in the graft loss group was significantly higher than the graft survival group (0.146 versus 0.076, p<0.001) (Figure 5A).

ROC curve analysis showed that the prediction score for dmtDNA levels was 0.779 (95% CI 0.682-0.876, p<0.001) (Figure 5B). According to the maximum Youden-J indices, the optimal prediction score cutoff value of the dmtDNA was ≥0.092, with the sensitivity and specificity of 80% and 69.5%, respectively. We also conducted ROC curve analysis to obtain the prediction score for the donor admission SCr and terminal SCr levels; the terminal SCr level (AUC=0.656, 95% CI 0.537-0.774, p=0.024) showed lower diagnostic value compared with the dmtDNA level (Figure 5C), but the donor admission SCr level showed no statistically significant difference for prediction of graft survival (AUC=0.602, 95%CI 0.470-0.734, p=0.139) (Figure 5D).

**Figure 6:**
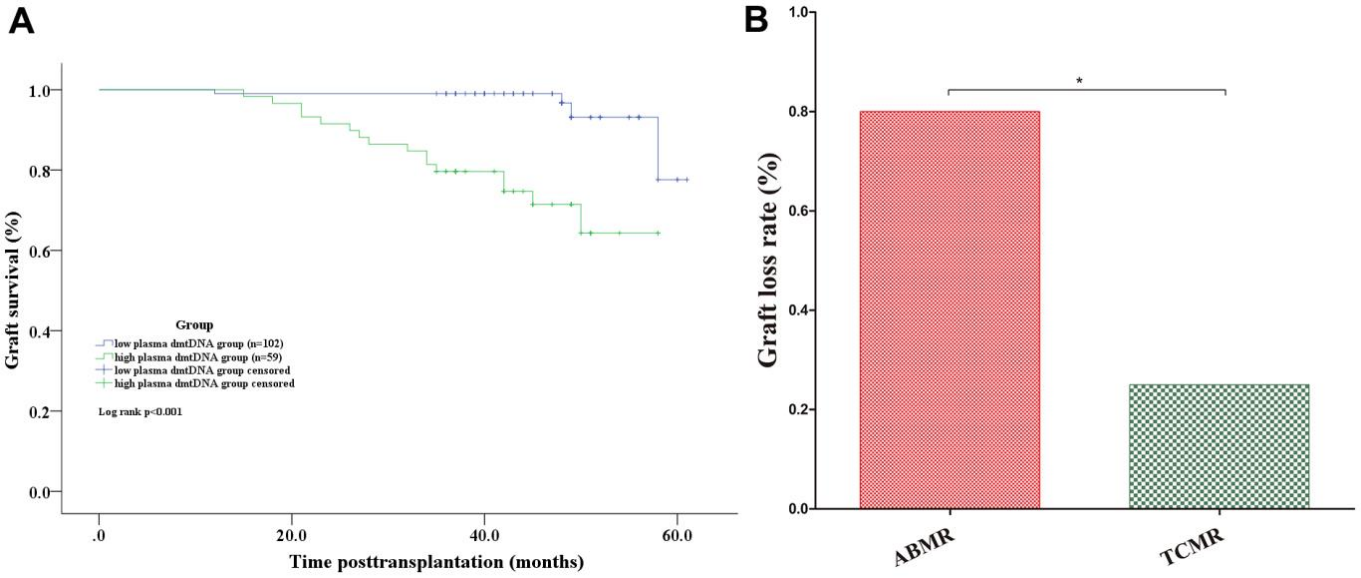
(**A**) Graft survival analyses. Kaplan-Meier curves for graft survival according to whether the donor plasma dmtDNA level was >0.092. (**B**) The graft loss rate in the ABMR group was significantly higher than that of the TCMR group. ABMR: antibody-mediated rejection; TCMR: T cell-mediated rejection.

We then divided the recipients into 2 groups according to the cutoff value of 0.092, and categorized the two groups as the low plasma dmtDNA group (n=102) and the high plasma dmtDNA group (n=59). Figure 6A shows that low dmtDNA group was with longer allograft survival time (96.1% versus 72.9%, p<0.001).

Among the recipients who lost the allograft, 50% were diagnosed with AR. Of the 10 recipients diagnosed with ABMR, 8 recipients lost the allograft. However, of the 8 recipients diagnosed with TCMR, only 2 recipients lost their allografts (80% versus 33.3%, p=0.031) (Figure 6B).

## DISCUSSION

As far as we know, this is the first study to elaborate the association of dmtDNA levels with ABMR. dmtDNA have been suggested as a potentially sensitive biomarker for monitoring ABMR and graft survival post-transplantation. The higher the dmtDNA level, the greater the probability of ABMR and the shorter the survival time of the allograft.

Consistent with our previous findings, the dmtDNA level is an effective indicator for predicting the occurrence of DGF after transplantation. When certain organs are damaged, mtDNA can be released from injured cells into circulation [14, 15]. A number of studies have shown that circulating mtDNA is elevated in various disease conditions, including diabetic retinopathy [16], AKI [17], trauma [18], and patient mortality in the intensive care unit [19]. DGF is a common complication following a transplant procedure, and is associated with a negative impact on clinical outcomes. A systematic review showed that recipients with DGF had a 41% increased risk of graft loss [20]. Our previous study also found that DGF is an independent risk factor for graft survival in recipients with ECD kidneys [21]. mtDNA levels have been confirmed as a predictive marker of AKI, and correlate with severity of renal dysfunction in chronic kidney injury [22]. In this study, dmtDNA confirmed as an independent risk factor for DGF. When the dmtDNA level was >0.133, the probability of DGF in the recipients was 50%. Another study has shown that urinary mtDNA levels are significantly associated with DGF short-term post-transplant renal function, acute rejection of the graft biopsy [23]. Therefore, dmtDNA could be a new noninvasive predictor of DGF, this finding is of great significance for application in the clinical setting.

Plasma dmtDNA levels may be the first biomarker that can reliably predict AR prior to undergoing a transplant. The use of immunosuppressive therapy after kidney transplantation has significantly improved allograft survival, but nearly 50% recipients will lose their allograft within 10 years due to AR [2, 4, 5]. A study that included 315 allograft recipients who underwent indication biopsies found that 64% of recipients lost their allografts due to AR [4]. In this study, a total of 18 (11.2%) recipients diagnosed with AR, with the average follow-up time for the 161 recipients being 43.5±8.5 months. The incidence of AR in this study is similar to that reported in other studies [24, 25]. When the dmtDNA level was > 0.0748, the probability of AR was 23.5%. However, when the dmtDNA level was >0.156, the probability of AR was 62.9%. Result showed that the dmtDNA level was an independent risk factor for AR, when the dmtDNA level was >0.0748, the odds ratio for AR was 1.588. Similar to the findings in this study, another recent study showed that donor-derived cell-free DNA levels were increased in the recipient’s blood when the allograft had developed AR [26]. We believe that the dmtDNA level has a higher predictive value than that of cell free DNA, and is a reliable predictive marker prior to transplantation, which lends the dmtDNA level greater clinical value. Many factors that can lead to AR, including HLA mismatch and cold ischemia time. Studies have shown that that DGF is an important risk factor for AR [27]. When the kidney is damaged, plasma dmtDNA levels increase, which may be accompanied by more antigen exposure, resulting in increased AR incidence. In contrast, increased dmtDNA levels may not only trigger inflammation, but may also lead to AR [28, 29]. Elevated dmtDNA levels are an independent risk factor not only for DGF, but also for AR.

Interestingly, the diagnostic value of the dmtDNA level for predicting ABMR is notably higher than for AR and TCMR. Of the 18 recipients diagnosed with AR, 10 were diagnosed with ABMR, and 8 were diagnosed with TCMR. The level of dmtDNA in the ABMR group was significantly higher than TCMR group. The prediction score of the dmtDNA level for ABMR was up to 0.910. When the dmtDNA level was >0.185, the probability of ABMR was 23.5%. Most instances of ABMR occurred one year after transplantation, whereas TCMR occurred within one year. There are many factors that contribute to TCMR, but the main reason is the lack of immunosuppressive therapy in the early postoperative period. Early TCMR responds well to treatment [30], and it is therefore believed that TCMR can be reversed and stabilized [31]. ABMR can arise for a variety of reasons, such as a history of transplantation, blood transfusions, or polytocous pregnancy [32, 33]. Donor-specific antibodies play pivotal roles during ABMR, and the incidence of DSA ranges from 20 to 30% post-transplantation [34]. The allograft function of recipients in the high-level dmtDNA group may be more susceptible to other damage such as drugs toxicity, which increases the probability of antigen exposure, thereby increasing the probability of DSA and development of ABMR. Therefore, we recommend that patients with high levels of dmtDNA should receive increased treatment with immunosuppressive drugs after transplantation.

Our study has several limitations that should be noted. This is a retrospective cohort study, and the number of recipients with AR is relatively low. This will have some impact on the results and conclusions of the study. To address this limitation, we are carrying out a prospective randomized multi-center clinical trial and have registered on the Chinese Clinical Trial Registry in order to verify our results from this study.

We conclude that the plasma dmtDNA level may be the first biomarker identified as a reliable predictor of AR prior to transplantation, especially for development of ABMR. dmtDNA is correlated with graft survival.

## MATERIALS AND METHODS

### Study design

This was a retrospective open cohort study that enrolled 167 donors and 323 recipients from January 2015 to December 2017 at the Third Affiliated Hospital of Sun Yet-sen University. The exclusive criteria of donors were as following: (1) did not have serum samples, (2) were <16 years of age at the time donation, (3) provided serum samples were damaged, (4) had multiple injuries. The exclusive criteria of recipients were as following: (1) the allograft was primary nonfunction, (2) died with functional grafts, (3) withdrew consent. This study was approved by the Human Organ Transplantation and Ethics Committee of Sun Yat-sen University [13]. All experimental procedures were described in our previous study [13, 35, 36].

### Data sources

We obtained most of donor data from COTRS, and some patient information was obtained from OPO’s charts. ECD donors were defined as being older than 60 years and donors aged 50-59 years with two of the following three characteristics: (1) cerebrovascular accident as cause of death, (2)history of systemic hypertension, and (3) terminal serum creatinine concentration >1.5 mg/dL [37].

We obtained recipient data from medical records, and early graft function after transplantation was classified as immediate graft function (IGF) and delayed graft function (DGF). DGF was defined as recipients who presented with a creatinine reduction ratio (CRR) ≤70% and required dialysis from day 0 to day 7 post-transplantation. IGF was defined as recipients who presented with a CRR >70% one week post-transplantation [38].

Donor blood samples were obtained from donors before organ procurement as previously described. The samples were treated as our previous study described [13].

### Measurement of relative donor mitochondrial DNA levels

As described in previous studies, plasma mtDNA levels were determined via quantitative real-time polymerase chain reaction (qPCR) [13, 39, 40]. All samples were assayed in a 96-well plate using a SLAN-96S Real-Time PCR System (ZEESAN, Xiamen, China). The same negative control and calibrator cDNA samples were used for each plate, and the samples were reanalyzed if the cycle threshold (Ct) value was >35 or the amplification curve was abnormal.

### Histopathology

Recipient underwent indication biopsies serum when creatinine increase >20% from baseline. Surveillance biopsies were performed at 3 months post-transplantation, unless the patient had surgical contraindications. For allograft punctures, 18-gauge needles were used and obtained 1-2 cores for pathological testing. All biopsies were processed and analyzed by the pathology laboratory, according to the Banff criteria for preparation of paraffin-embedded sections and grading [41]. C4d-negative Antibody-mediated rejection (ABMR) was defined as: positive for donor-specific antibodies (DSA), non-diffuse C4d staining, and any of the following microcirculation lesions: glomerulitis (g > 0), thromboses and transplant glomerulopathy (cg > 0), or peritubular capillaritis (ptc >0) [42].

### Statistical analysis

The Mann-Whitney U test was used to compare the differences between two independent groups for nonnormally distributed variables, and Student’s t test was used to test whether there is a difference between two groups on a continuous dependent variable. Pearson Chi-square test or Fisher’s exact test was used to assess the percentage for categorical variables.

The diagnostic value of dmtDNA and serum creatinine levels with respect to prediction of AR, ABMR, and graft survival were evaluated in ROC curve analyses. Youden’s index is often used in conjunction with ROC analysis [43]. Statistical analysis was carried out using the IBM SPSS statistics version 20.0.
